# How Much Morphine Can a Dog Stand? Can Arsenic Produce Paralysis of the Lower Jaw?

**Published:** 1891-01

**Authors:** John A. McLaughlin

**Affiliations:** Providence, R. I.


### HOW MUCH MORPHINE CAN A DOG STAND? CAN ARSENIC PRODUCE PARALYSIS OF THE LOWER JAW?

JOHN A. McLAUGHLIN, D. V. S.,
PROVIDENCE, R. I.

THESE are two questions I would like to hear discussed by members of the profession. The first question has arisen in my mind from the enormous amount I have given my own dog, a small collie, weighing probably twenty-five or thirty pounds, and the latter by three cases which have come under my professional observation, of which I will speak later.

My collie is about fifteen months old, quite a small one, and showed symptoms of acute gastritis due, I supposed, to a foreign (non-poisonous) body; she began whining and crying, became extremely restless, breathing short, mouth partly open and the mucous membranes of that organ injected. Large doses of oil, ipecac and opium succeeded, in the course of a week, in removing all symptoms, and when they remained absent for four or five days I felt justified in giving her a run. She was exercised for two successive days, and on the second the symptoms returned, not caused by exercise, I believe, but by another similar cause as her previous attack. The symptoms were the same, but much severer. I administered one-fourth grain of morphine without effect, ten or fifteen minutes later I gave one-half grain, and within fifteen minutes after the second dose I gave one and one-fourth grains, without a particle of effect. I bought an one-eighth ounce bottle, and making a solution of about two grains I injected it hypodermically. This quieted her. Twice during the night, and every time she began to whine and cry during seventy-two hours, I kept this treatment up, and found by actual weight I had fifteen grains injected subcutaneously; in the next twenty-four hours I administered four grains, per mouth, in one grain doses. My hypodermic syringe allowed some loss, and for this I will say instead of fifteen grains my dog received but ten grains, which amount she surely got. Here is a record of ten grains hypodermically and six grains per mouth in four days, and with only good, yes, wonderful results. Here was a dog with acute gastritis so severe as to render her countenance pitiful to behold, so truly did it express the intense agony she was suffering; the buccal mucous membranes injected to a dirty, nasty, dull brickish color, the tongue wrinkled, shrunken and parched, the breath horribly fetid, without any hope of recovery that I could see, showing the most favorable symptoms twenty-four hours after the last dose of morphine.

In the course of the last six months I have attended three cases of paralysis of the lower jaw. All three were brought to me under impression they had something lodged in the throat. My diagnosis of each was gastritis due to arsenical poisoning. My diagnosis in the first was verified by an examination by Dr. Heaton, who assisted me in the post-mortem; the specimens of the second were lost; and the stomach and intestines, and their contents, of the third are now under observation.

According to our authorities, paralysis of the lower jaw *is a sure sign of dumb rabies in the canine*, and Dr. Hill, who is an excellent and a clever canine practitioner, has held to the spontaneous character of the disease, mostly from this one symptom. Would it not be curious if *dumb rabies*, so called, was due very often to those thousand and one abominations, or godsends, whichever way you put it, with which we exterminate rats and other vermin, and which have, as a rule, arsenic as their exterminating base?

I would like very much to hear the opinion of the profession on the subject.

